# Preoperative Plasma and Cerebrospinal Fluid SNAP‐25 Levels Predict Delayed Neurocognitive Recovery in Elderly Patients: A Prospective Observational Study

**DOI:** 10.1002/cns.71028

**Published:** 2026-07-09

**Authors:** Xiao‐Yu Dou, Jing‐Yu Li, Yi Qiu, Meng‐Ying Xu, Hai‐Yun Dong, Jiao Xue, Peng‐Lei Ma, Min An

**Affiliations:** ^1^ Inner Mongolia Medical University Hohhot China; ^2^ The Second Affiliated Hospital Inner Mongolia Medical University Hohhot China

**Keywords:** cerebrospinal fluid, delayed neurocognitive recovery, elderly, plasma, synaptosomal‐associated protein 25

## Abstract

**Objective:**

Delayed neurocognitive recovery (DNR) is a common postoperative complication in elderly patients, and its prevention and treatment remain challenging. Synaptosomal‐associated protein 25 (SNAP‐25), a potential biomarker for several neurodegenerative diseases, has been reported to be associated with the occurrence of DNR. However, whether SNAP‐25 can predict the development of DNR remains unclear. Therefore, this study aimed to investigate the predictive value of preoperative SNAP‐25 levels in cerebrospinal fluid (CSF) and plasma for DNR in elderly patients.

**Methods:**

In this prospective observational study, elderly patients aged 65–75 years undergoing total knee arthroplasty under spinal anesthesia were enrolled. 3 mL of venous blood was collected upon entry into the operating room, and 1 mL of CSF was obtained after successful lumbar puncture and prior to intrathecal anesthetic injection. Neurocognitive function was assessed 1 day before surgery and 7 days after surgery. The primary outcome was the incidence of DNR. The predictors were preoperative CSF and plasma SNAP‐25 levels. The association between SNAP‐25 levels and DNR was further evaluated.

**Results:**

A total of 86 patients completed the study, among whom 21 developed DNR, yielding an incidence of 24.4%. Preoperative SNAP‐25 levels in both CSF and plasma were significantly lower in the DNR group than in the non‐DNR group. Given the correlation between CSF and plasma SNAP‐25 levels (*r* = 0.89), two separate multivariable logistic regression models were constructed to avoid multicollinearity, higher SNAP‐25 levels were associated with a reduced risk of DNR. ROC analysis showed that CSF SNAP‐25 had an AUC of 0.805 (95% CI: 0.706–0.905), with an optimal cutoff value of 737.5 pg/mL (sensitivity: 0.905; specificity: 0.569), whereas plasma SNAP‐25 had an AUC of 0.727 (95% CI: 0.618–0.837), with an optimal cutoff value of 543.5 pg/mL (sensitivity: 0.714; specificity: 0.708).

**Conclusion:**

Preoperative SNAP‐25 in both CSF and plasma is associated with DNR; although CSF SNAP‐25 shows better discrimination, plasma SNAP‐25 offers more balanced performance and greater clinical feasibility as a practical biomarker for identifying at‐risk patients.

**Trial Registration:**

ChiCTR2500113164

## Graphical abstract

Preoperative SNAP‐25 levels in CSF and plasma were measured in elderly patients undergoing total knee arthroplasty, and DNR was assessed using neuropsychological tests. Lower SNAP‐25 levels predicted postoperative DNR. CSF SNAP‐25 showed better discrimination, whereas plasma SNAP‐25 demonstrated more balanced performance and greater clinical feasibility.

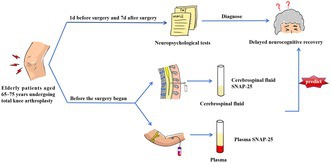

## 1 Introduction

Delayed neurocognitive recovery (DNR) is a common central nervous system complication following anesthesia and surgery, characterized by impairments in memory, information processing, and attention, often accompanied by emotional and personality changes [1]. Advanced age is one of the most important risk factors for the development of DNR [2]. Previous studies have reported that the incidence of DNR ranges from 5% to 55% in elderly patients undergoing noncardiac surgery and from 20% to 50% in those undergoing cardiac surgery [3]. DNR has been associated with increased mortality, prolonged hospitalization, and a higher long‐term risk of developing Alzheimer's disease (AD) [1, 4]. Consequently, DNR represents a major challenge for postoperative recovery in elderly patients. Early prediction and timely intervention may help reduce its occurrence; however, reliable and easily applicable predictive methods are still lacking in clinical practice. Therefore, identifying effective early predictors of DNR is of great clinical importance.

Synaptosome‐associated protein 25 (SNAP‐25) is an important protein involved in neuronal development, mediating synaptic vesicle fusion, exocytosis, and neurotransmitter release in neurons [5]. Previous studies have shown that the expression levels of SNAP‐25 in cerebrospinal fluid (CSF) and plasma are significantly altered in patients with neurodegenerative diseases, such as AD and Creutzfeldt–Jakob disease [6, 7]. Notably, decreased levels of SNAP‐25 in CSF have been detected 5–7 years before the onset of cognitive impairment in patients with AD, suggesting that SNAP‐25 can serve as a predictive biomarker for AD [8]. In addition, experimental studies in aged mice with postoperative cognitive dysfunction have also demonstrated altered expression of SNAP‐25 in the hippocampus following anesthesia and surgery [9]. However, whether SNAP‐25 levels can predict the occurrence of DNR in elderly patients remains unclear. Therefore, the present study aimed to investigate the predictive value of preoperative SNAP‐25 levels in CSF and plasma for postoperative DNR in elderly patients, providing a potential new approach for the early prevention of DNR.

## 2 Materials and Methods

### 2.1 Patients

This study was approved by the Ethics Committee of the Second Affiliated Hospital of Inner Mongolia Medical University (Approval No. EFY20250101(06)), and written informed consent was obtained from all patients or their legal representatives prior to enrollment. Patients scheduled for elective unilateral total knee arthroplasty under spinal anesthesia at the Second Affiliated Hospital of Inner Mongolia Medical University between July 2025 and October 2025 were recruited. Inclusion criteria were: (1) willingness to participate and provision of written informed consent; (2) age between 65 and 75 years, regardless of sex; (3) American Society of Anesthesiologists (ASA) physical status II or III; (4) preoperative Mini‐Mental State Examination (MMSE) score ≥ 24 on the day before surgery, with no speech or communication disorders and sufficient education to complete cognitive function testing. Exclusion criteria were: (1) history of neurological disorders, including AD, Parkinson's disease, head trauma, epilepsy, or multiple sclerosis; (2) inability to cooperate with preoperative MMSE assessment; (3) long‐term use of opioids or psychotropic medications; (4) contraindications to spinal anesthesia; (5) history of major surgery within the past 6 months; (6) patients discharged within 7 days after surgery.

### 2.2 Clinical Data Collection

On the day before surgery, all participants were interviewed to collect baseline data, including sex, age, educational level, and corrected body mass index (BMI). The corrected BMI was defined as BMI calculated based on the height at 40 years of age rather than the current height [10]. Potential perioperative confounding factors, such as duration of surgery, intraoperative blood loss, volume of fluid infusion, visual analog scale (VAS) scores, and patient‐controlled analgesia (PCA) pump usage, were also recorded.

### 2.3 Sample Collection and Processing

Venous blood (3 mL) was collected from the antecubital vein by a registered nurse prior to anesthesia induction in the operating room. After successful spinal puncture, CSF (1 mL) was collected before the administration of anesthetic agents. All samples were processed within 2 h: centrifuged at 2000 × g for 10 min, and the supernatants were aliquoted and stored at −80°C in enzyme‐free tubes. Plasma and CSF concentrations of SNAP‐25 were measured using enzyme‐linked immunosorbent assay (ELISA) kits (Human SNAP‐25 ELISA Kit, Jiangsu Yimmuno Industrial Co. Ltd.), and all procedures were performed strictly according to the manufacturer's instructions.

### 2.4 Neurocognitive Assessment

A series of neuropsychological tests were conducted preoperatively (one day before surgery) and postoperatively (seven days after surgery) in a quiet room with only the patient and the assessor present. The test battery included the MMSE, Digit Span Test (DST), Stroop Color Word Test (SCWT), Trail Making Test (TMT), and Clock Drawing Test (CDT). Patients with preoperative MMSE scores < 24 were excluded. Using healthy elderly individuals as a reference population, the practice effects were derived. After correcting for practice effects, *Z*‐scores for each test were calculated, and the mean of all test *Z*‐scores was used as the composite *Z*‐score [11]; the composite *Z*‐score > 1.96 was diagnosed as DNR. Participants were then divided into the DNR group (D group) and the non‐DNR group (ND group) according to the occurrence of DNR.

### 2.5 Anesthesia Protocol

All patients underwent standardized preoperative preparation, which included no premedication, fasting for 8 h, and no fluid intake for 6 h prior to surgery. Upon arrival in the operating room, electrocardiography (ECG), noninvasive blood pressure (BP), and peripheral oxygen saturation (SpO_2_) were monitored. Ultrasound‐guided femoral nerve block was performed on the operative side using 20 mL of 0.2% levobupivacaine. Spinal anesthesia was then administered with the patient in the lateral decubitus position (operative side up) at the L3‐L4 or L2‐L3 interspace. After successful puncture, 1 mL of CSF was first withdrawn, followed by injection of 2.5–3 mL of 0.6% ropivacaine over approximately 30 s, with the sensory block level maintained below T8. Intraoperatively, oxygen was administered via mask at 3 L/min, and blood pressure was maintained within 20% of baseline. Hypotension, defined as a decrease > 20% from baseline or < 90 mmHg, was treated with intravenous ephedrine 6 mg. Postoperative analgesia was provided using patient‐controlled intravenous analgesia (PCIA) with a solution of 120 μg nalbuphine and 10 mg tropisetron diluted to 100 mL with normal saline, targeting a VAS score < 4. All procedures were performed by the same team of anesthesiologists and surgeons to minimize inter‐operator variability.

### 2.6 Blinding Procedure

Assessors were provided only with basic demographic information, such as age and sex, and were blinded to surgical type, anesthesia method, and intraoperative time points that could reveal the surgical status. Assessors were unaware of the timing sequence of evaluations and completed neurocognitive assessments solely based on patient performance during testing. To evaluate the integrity of blinding, consistency checks and surveys of assessor knowledge were conducted. If minor bias was detected, stratified analysis or propensity score matching was applied; data were excluded if blinding failure resulted in significant bias. ELISA measurements were performed independently by two laboratory technicians. After completion of all assays, a blinding assessment questionnaire was administered to the technicians. The accuracy of their guesses was compared with the 50% probability expected by chance, and a binomial test was used to evaluate the success of blinding.

### 2.7 Statistical Analysis

Data distribution guided the presentation of continuous variables (mean ± SD or median with IQR) and categorical variables (counts and percentages). For group comparisons, continuous data were analyzed using Student's *t*‐test, Mann–Whitney *U* test, or Kruskal–Wallis test as appropriate, while categorical data were compared using the chi‐square test or its continuity‐corrected version. Normality and variance homogeneity were checked using the Kolmogorov–Smirnov and Bartlett's tests, respectively. To identify predictors of DNR, multivariable logistic regression analysis was performed. Given the correlation between preoperative CSF and plasma SNAP‐25 levels (*r* = 0.89, *p* < 0.001), two separate multivariable models were constructed to avoid multicollinearity and ensure model stability. Possible risk factors for impaired postoperative cognitive recovery as reported in prior studies, including age, educational level, duration of surgery, blood loss, and postoperative pain [12, 13, 14, 15], were included in the models. Results are presented as odds ratios (ORs) with 95% confidence intervals (CIs).

The predictive performance of preoperative plasma and CSF SNAP‐25 levels for DNR was evaluated using receiver operating characteristic (ROC) curve analysis, with calculation of the area under the curve (AUC), optimal cutoff value, sensitivity, and specificity. The optimal cutoff values were determined using the maximum Youden index. The DeLong test was used to compare the AUCs between plasma and CSF SNAP‐25. To assess the stability of model performance, bootstrap resampling with replacement was performed 1000 times (*n* = 86 per resample) to estimate the mean AUC and its 95% percentile confidence interval for both plasma and CSF SNAP‐25. DeLong test and bootstrap analyses were performed using R software (version 4.5.3), while all other statistical analyses were conducted using IBM SPSS Statistics version 25.0 (IBM, Armonk, NY). With *p* < 0.05 considered statistically significant.

### 2.8 Sample Size

This was a single‐center prospective observational study. The sample size was determined based on both statistical considerations and clinical feasibility. According to studies in Chinese populations, the incidence of DNR on postoperative day 7 after total knee arthroplasty is approximately 23.6%–24% [16, 17]. Based on these findings, the expected incidence was set at 25%. According to the standard formula for estimating a single population proportion. Assuming an expected incidence of DNR of 25%, a margin of error of 0.1, and a 95% confidence level (corresponding to μ = 1.96), the calculation yielded a minimum required sample size of 72 patients. Considering that approximately 120–150 eligible patients undergo surgery at our center within a single quarter, and accounting for an anticipated dropout or incomplete data rate of 10%–15%, a total of 110 patients were initially enrolled.

## 3 Results

### 3.1 Patient Characteristics

**FIGURE 1:**
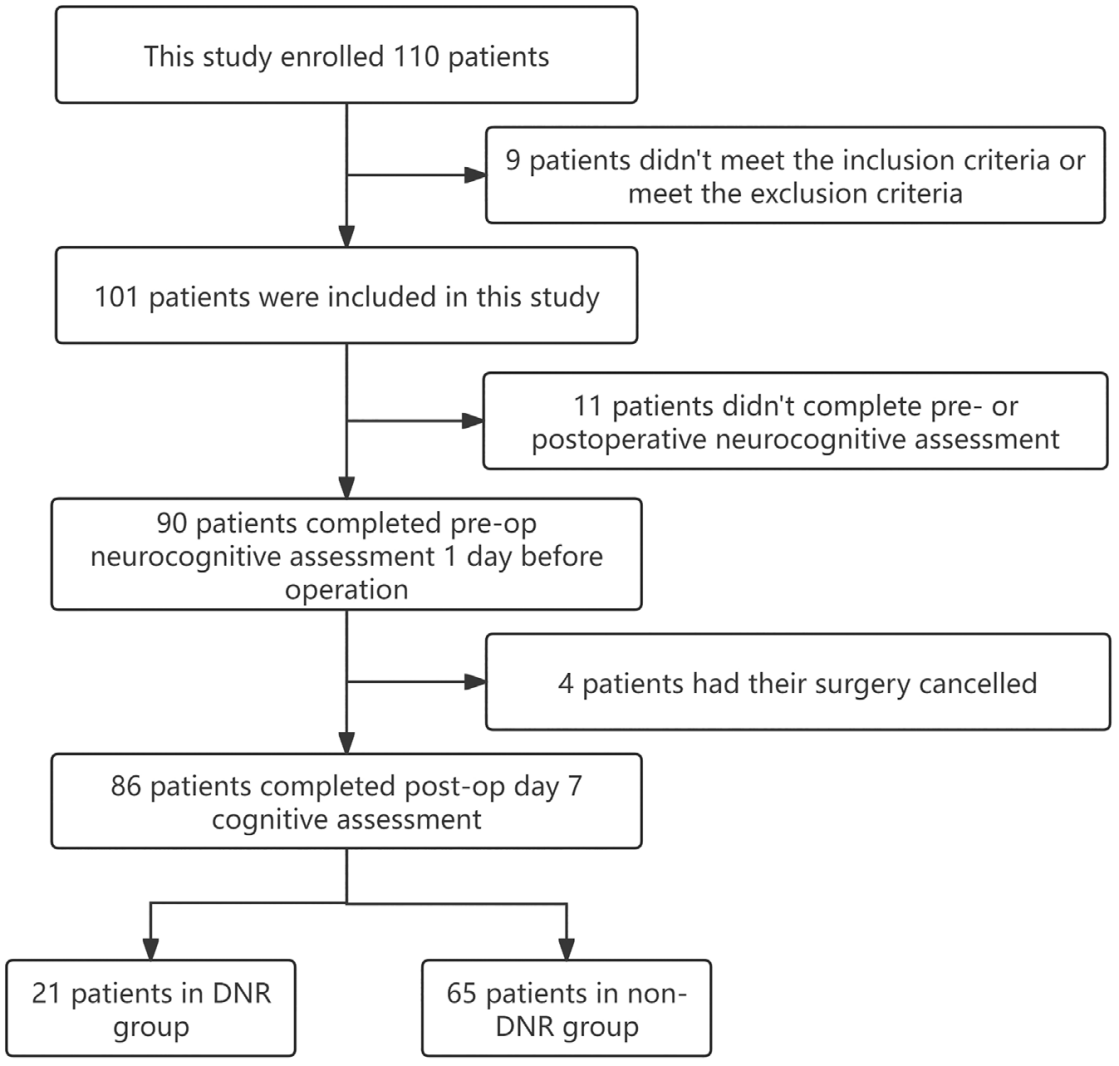
Flowchart of the study procedure. DNR, delayed neurocognitive recovery.

A total of 110 patients were enrolled in this study, of whom 86 completed the entire trial (Figure 1). Baseline characteristics did not differ significantly between completers and noncompleters of the entire trial. Based on neurocognitive assessments, 21 patients developed DNR, while 65 patients did not, resulting in a DNR incidence of 24.4%. Baseline characteristics and perioperative confounding factors for the D group and ND group are summarized in Table 1. No significant differences were observed between the two groups with respect to sex, age, BMI, educational level, duration of surgery, VAS scores and others (Table 1).

**TABLE 1:** Clinical characteristics of patients in the DNR and non‐DNR groups.

Variable	*D* group (*n* = 21)	ND group (*n* = 65)	*p*
Male, *n* (%)	5 (23.8)	16 (24.6)	0.940
Age (years), median (IQR)	72.0 (69.5–73.0)	71.0 (69.0–72.5)	0.378
BMI (kg/m^2^), mean ± SD	27.10 ± 3.44	26.09 ± 3.01	0.198
Educational level (higher than secondary school), *n* (%)	3 (14.3)	16 (24.6)	0.491
Hypertension, *n* (%)	6 (28.6)	13 (20.0)	0.603
Diabetes, *n* (%)	3 (14.3)	18 (27.7)	0.214
Duration of surgery (min), median (IQR)	90.0 (82.5–100.0)	90.0 (67.5–105.0)	0.960
Infusion volume (mL), median (IQR)	1200 (1100–1300)	1200 (1000–1275)	0.093
Blood loss (mL), median (IQR)	110 (110–155)	120 (100–150)	0.778
PCA activations (times), median (IQR)	8 (7–9)	8 (7–9)	0.776
VAS, median (IQR)	1 (1–2)	1 (1–2)	0.767
Pre‐op MMSE, median (IQR)	27.0 (26.0–28.0)	27.0 (25.5–28.0)	0.771
Pre‐op SCWT (s), median (IQR)	43.0 (29.5–58.0)	38.0 (33.0–47.5)	0.577
Pre‐op CDT, median (IQR)	4 (3–4)	4 (3–4)	0.438
Pre‐op TMT (s), median (IQR)	156.0 (90.5–200.0)	115.0 (89.5–150.5)	0.168
Pre‐op DST, median (IQR)	8.0 (6.5–9.0)	8.0 (7.0–9.0)	0.699
Composite *Z*‐score, median (IQR)	0.26 (0.07–0.46)	2.63 (2.33–3.18)	< 0.001***
CSF SNAP‐25 (pg/mL), mean ± SD	679.3 ± 53.3	756.5 ± 68.0	< 0.001***
Plasma SNAP‐25 (pg/mL), mean ± SD	522.6 ± 55.7	584.2 ± 79.2	0.001**

*Note:* The presentation of continuous variables (mean ± SD or median with IQR) and categorical variables (counts and percentages). For group comparisons, continuous data were analyzed using Student's *t*‐test, Mann–Whitney *U* test, or Kruskal–Wallis test as appropriate, while categorical data were compared using the chi‐square test or its continuity‐corrected version. *p* ** means *p* value < 0.01; *** *p* means *p* value < 0.001. *D* group is defined as patients who developed DNR; ND group is defined as patients who did not develop DNR. Preoperative hypertension was defined according to the following thresholds: systolic pressure ≥ 140 mmHg or diastolic pressure ≥ 90 mmHg. For diabetes mellitus, diagnostic criteria included a fasting plasma glucose ≥ 7.0 mmol/L or a 2‐h postprandial glucose ≥ 11.0 mmol/L.

Abbreviations: BMI, body mass index; CDT, Clock Drawing Test; CSF, cerebrospinal fluid; DNR, delayed neurocognitive recovery; DST, Digit Span Test; MMSE, Mini‐Mental State Examination; PCA, patient‐controlled analgesia; SCWT, Stroop Color Word Test; SNAP‐25, synaptosomal‐associated protein 25; TMT, Trail Making Test; VAS, visual analog scale.

Neuropsychological testing revealed no significant differences between the groups in preoperative cognitive assessments (Table 1). On postoperative day 7, the D group exhibited significantly poorer performance compared to the ND group, with postoperative MMSE, SCWT, CDT, and DST scores showing significant differences (*p* < 0.05). No significant difference was observed in TMT between the two groups (Supplementary Table S1).

### 3.2 Relationship Between Preoperative CSF and Plasma SNAP‐25 Levels and DNR

Compared with the ND group, patients in the D group had significantly lower preoperative CSF and plasma SNAP‐25 levels (Table 1). To identify the optimal biomarker for predicting DNR, preoperative CSF and plasma SNAP‐25 levels were further analyzed. Multivariable logistic regression analysis showed that higher preoperative SNAP‐25 levels in both plasma and CSF were associated with a reduced risk of DNR. In the CSF model, each 1 pg/mL increase in SNAP‐25 was associated with a 7.6% decrease in the odds of DNR (OR = 0.924, 95% CI: 0.888–0.962, *p* < 0.001). In the plasma model, each 1 pg/mL increase in SNAP‐25 was associated with a 2.3% decrease in the odds of DNR (OR = 0.977, 95% CI: 0.964–0.991, *p* = 0.001) (Table 2).

**TABLE 2:** Multivariable logistic regression analysis of risk factors for DNR.

Models	CSF		Plasma	
	OR (95% CI)	*p*	OR (95% CI)	*p*
Model 1	0.979 (0.968–0.990)	< 0.001***	0.988 (0.979–0.996)	0.003**
Model 2	0.968 (0.952–0.984)	< 0.001***	0.985 (0.976–0.995)	0.003**
Model 3	0.937 (0.908–0.968)	< 0.001***	0.978 (0.965–0.991)	0.001**
Model 4	0.924 (0.888–0.962)	< 0.001***	0.977 (0.964–0.991)	0.001**

*Note:* Model 1 is unadjusted; Model 2 is adjusted for preoperative variables (age, educational level); Model 3 is adjusted for intraoperative variables (duration of surgery, blood loss); Model 4 is adjusted for postoperative variables (VAS). *p* ** means *p* value < 0.01; *p* *** means *p* value < 0.001.

Abbreviations: CI, confidence interval; CSF, cerebrospinal fluid; CSF, cerebrospinal fluid; DNR, delayed neurocognitive recovery; OR, odds ratio; PCA, patient‐controlled analgesia; VAS, visual analog scale.

**FIGURE 2:**
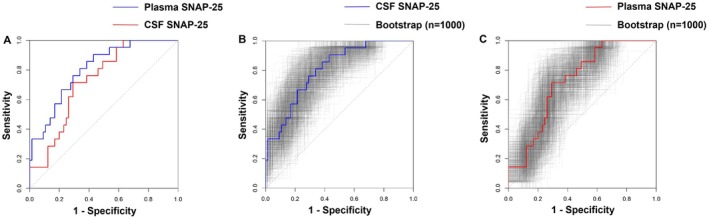
(A) ROC curves of SNAP‐25 levels in plasma and CSF. (B) ROC curve of CSF SNAP‐25 for predicting DNR based on bootstrap analysis. (C) ROC curve of plasma SNAP‐25 for predicting DNR based on bootstrap analysis. CSF, cerebrospinal fluid; ROC, receiver operating characteristic; SNAP‐25, synaptosomal‐associated protein 25.

ROC curve analysis showed that the AUC of CSF SNAP‐25 was 0.805 (95% CI: 0.706–0.905), with an optimal cut‐off value of 737.5 pg/mL (sensitivity: 0.905; specificity: 0.569). The AUC of plasma SNAP‐25 was 0.727 (95% CI: 0.618–0.837), with an optimal cut‐off value of 543.5 pg/mL (sensitivity: 0.714; specificity: 0.708) (Table S2, Figure 2A). The DeLong test indicated a statistically significant difference between the AUCs of CSF and plasma SNAP‐25 (*p* = 0.007). Bootstrap analysis demonstrated that, based on 1000 resampling iterations, the mean AUC was 0.794 (95% CI: 0.693–0.889) for CSF SNAP‐25 and 0.719 (95% CI: 0.607–0.826) for plasma SNAP‐25, indicating good stability of both predictive models (Table S3, Figure 2B,C).

Subsequently, patients were divided into high and low groups according to the cutoffs for CSF SNAP‐25 (> 737.5 vs. ≤ 737.5 pg/mL) and plasma SNAP‐25 (> 543.5 vs. ≤ 543.5 pg/mL). The low CSF SNAP‐25 group showed a significantly higher incidence of DNR than the high CSF SNAP‐25 group (*p* < 0.001), and similarly, the low plasma SNAP‐25 group had a higher DNR incidence than its high counterpart (*p* = 0.004) (Tables S4 and S5).

## 4 Discussion

DNR is a common postoperative complication in elderly patients. Currently, the diagnosis of DNR mainly relies on neuropsychological testing [18]. However, the definition and assessment tools used to evaluate DNR vary across studies [19], which may result in delayed or inaccurate identification and consequently limit the implementation of early prevention and intervention strategies. Therefore, the identification of stable, reproducible, and clinically applicable biomarkers is of particular importance. In this study, we found that SNAP‐25 levels in both plasma and CSF were significantly associated with the occurrence of DNR. After adjusting for common confounding factors, including age, educational level, duration of surgery, blood loss, and postoperative pain, this association remained statistically significant, suggesting that SNAP‐25 may serve as an independent correlate of DNR. Further analysis demonstrated that preoperative CSF and plasma SNAP‐25 levels had predictive value for the occurrence of postoperative DNR in elderly patients. The DeLong test showed a statistically significant difference between the AUCs of the two biomarkers (*p* = 0.007), with CSF SNAP‐25 demonstrating superior discriminative ability. However, at the optimal cutoff value of 737.5 pg/mL, CSF SNAP‐25 showed high sensitivity (0.905) but relatively low specificity (0.569), indicating strong screening performance but a relatively high false‐positive rate, which may lead to misclassification of patients without DNR as positive cases. In addition, CSF samples are obtained via lumbar puncture, an invasive procedure that limits its routine application in clinical screening. These factors reduce the clinical utility of CSF SNAP‐25 when used alone for prediction. In contrast, although plasma SNAP‐25 showed slightly lower overall predictive performance, it demonstrated a more balanced diagnostic profile at the optimal cutoff value of 543.5 pg/mL, with sensitivity (0.714) and specificity (0.708) being relatively well balanced. This suggests more stable classification performance and more reliable positive results. Moreover, the measurement is simple and reproducible. Therefore, plasma SNAP‐25 may be more suitable as a screening biomarker for DNR risk.

In earlier studies, DNR was generally classified as postoperative cognitive dysfunction (POCD). However, according to recent consensus recommendations, DNR has been redefined as a subtype of postoperative neurocognitive disorders (PND), referring to cognitive decline occurring within 30 days after surgery [20]. Therefore, in the literature cited in this study, the term “POCD” used in earlier publications has been interpreted according to the updated PND classification framework.

Previous studies have indicated that neuroinflammation in the central nervous system is one of the key pathological processes underlying DNR. Surgical procedures can induce activation of the NLRP3 inflammasome in astrocytes, subsequently triggering pyroptosis and ultimately contributing to the development of DNR [21]. In addition, in a mouse model of DNR, overexpression of tumor necrosis factor‐α (TNF‐α) in the hippocampus disrupts mitochondrial dynamics, whereas enhancement of mitochondrial function has been shown to improve cognitive performance [22]. These findings suggest that inflammation‐mediated pyroptosis and mitochondrial dysfunction may represent important components of the pathogenesis of DNR.

As a key component of the presynaptic SNARE complex, SNAP‐25 plays a central role in synaptic vesicle exocytosis, neurotransmitter release, and the maintenance of cognitive function [23]. Previous studies have demonstrated that decreased SNAP‐25 expression is closely associated with the severity of cognitive impairment in neurodegenerative diseases such as AD and dementia with Lewy bodies [24]. Given that DNR and AD share several pathological pathways, including synaptic dysfunction, neuroinflammation, and mitochondrial injury [25], alterations in SNAP‐25 levels may also be associated with the development of DNR.

Animal studies further support the association between SNAP‐25 and DNR. Wu et al. reported a significant downregulation of SNAP‐25 expression in the hippocampus of aged mice with DNR [26]. This phenomenon may be related to the postoperative upregulation of TNFAIP1, an E3 ubiquitin ligase that promotes the degradation of SNAP‐25 [27]. In cases of severe SNAP‐25 deficiency, although presynaptic active zones can still support vesicle docking, the readily releasable vesicle pool is markedly depleted, and rapid calcium‐triggered exocytosis is impaired, ultimately leading to neurotransmitter release deficits and synaptic dysfunction [28]. Moreover, recent evidence suggests that SNAP‐25 may exert neuroprotective effects by inhibiting caspase‐3/gasdermin E (GSDME)‐dependent pyroptosis and promoting mitophagy, thereby alleviating inflammation‐induced neuronal injury during the progression of DNR [29].

Consistent with these findings, our clinical study demonstrated that patients who developed DNR had lower preoperative SNAP‐25 levels. This observation suggests that the reduction in SNAP‐25 may occur before the onset of clinical symptoms. At the preoperative stage, patients who subsequently develop DNR may already exhibit subtle but significant decreases in SNAP‐25 levels, which could increase their vulnerability to surgery‐induced inflammatory responses. Therefore, decreased SNAP‐25 may not only represent a consequence of the pathological process of DNR but may also reflect the early initiation of key pathological events, supporting its potential role as a predictive biomarker for DNR.

Previous studies have demonstrated that SNAP‐25 can serve as a predictive biomarker for AD, although it is not specific to AD and lacks the ability to distinguish AD from other neurodegenerative disorders [25]. To minimize the potential confounding effects of neurodegenerative diseases on the study outcomes, patients with a history of psychiatric or neurodegenerative disorders (such as AD or Parkinson's disease) were excluded during enrollment. On this basis, we further compared the predictive performance of preoperative SNAP‐25 levels in plasma and cerebrospinal fluid. In this study, CSF SNAP‐25 demonstrated superior discriminative ability in predicting DNR. This may be explained by the fact that brain‐derived proteins in plasma are more susceptible to degradation by proteases, hepatic metabolism, and renal clearance, leading to unstable levels [30]. Additionally, the blood–brain barrier limits the entry of most brain‐derived proteins into plasma, making plasma levels less reflective of central synaptic injury [31]. In contrast, CSF is more closely associated with the brain microenvironment and may therefore more accurately reflect preoperative alterations in synaptic function, resulting in greater predictive value. However, after comprehensive consideration of diagnostic performance and clinical accessibility, plasma SNAP‐25 may be more suitable as a screening biomarker for DNR risk.

Early identification, prevention, and management of perioperative risk factors remain key strategies for reducing the incidence of DNR. Compared with postoperative biomarkers, preoperative indicators provide greater predictive value for risk stratification. The present findings suggest that lower preoperative SNAP‐25 levels may serve as an early warning signal before the onset of clinical symptoms, allowing surgeons, anesthesiologists, and perioperative care teams to identify high‐risk patients and implement individualized perioperative management strategies in advance. Risk stratification based on SNAP‐25 levels may also facilitate more precise anesthetic decision‐making. For example, prior studies have shown that total intravenous anesthesia is associated with a lower incidence of DNR compared with inhalation anesthesia [32], and neuraxial anesthesia carries a lower DNR risk than general anesthesia [33]. Therefore, for patients with low preoperative SNAP‐25 levels, anesthetic strategies with higher neuroprotective potential should be preferentially considered. Additionally, intraoperative hypothermia has been closely linked to DNR [34], highlighting the need for enhanced temperature management in patients with low SNAP‐25. Integrating SNAP‐25–based risk assessment with perioperative management strategies may further improve the early prevention of DNR.

Several limitations of this study should be acknowledged. First, this was a single‐center study with a relatively small sample size; therefore, multicenter studies with larger cohorts are needed to validate these findings. Second, only elderly patients undergoing unilateral knee arthroplasty were included, which may limit the generalizability of the results to other surgical populations. Third, this study mainly focused on the association between preoperative fluid SNAP‐25 levels and DNR and did not explore the causal mechanisms or temporal dynamics of SNAP‐25 alterations. Future studies are warranted to further elucidate the biological mechanisms underlying the involvement of SNAP‐25 in the development of DNR.

In conclusion, preoperative CSF and plasma SNAP‐25 levels were both associated with the occurrence of DNR in elderly surgical patients. Although CSF SNAP‐25 demonstrated superior discriminative ability, plasma SNAP‐25 showed a more balanced diagnostic performance and greater feasibility for routine clinical application. Therefore, plasma SNAP‐25 may serve as a practical screening biomarker for identifying patients at risk of DNR. These findings provide a new perspective for the early identification and risk stratification of DNR.

## Author Contributions

X.‐Y.D. and J.‐Y.L.: conceptualization, methodology, formal analysis, investigation, data curation, writing – original draft. Y.Q. and J.X.: investigation, resources, data curation, project administration. M.‐Y.X. and H.‐Y.D.: software, validation, formal analysis, visualization. M.A. and P.‐L.M.: conceptualization, resources, writing – review and editing, supervision, funding acquisition. All authors: Read and approved the final manuscript.

## Funding

Supported by the General Project of Inner Mongolia Medical University (YKD2023MS006) and the Clinical Medicine Research and Clinical New Technology Promotion Project of Inner Mongolia Medical Doctor Association (YSXH2024KYF061).

## Ethics Statement

The Ethics Committee of the Second Affiliated Hospital of Inner Mongolia Medical University, Approved No. of ethic committee: EFY20250101(06).

## Conflicts of Interest

The authors declare no conflicts of interest.

## Supporting information


**Table S1:** Neuropsychological test scores on postoperative day 7 in the DNR and non‐DNR groups.
**Table S2:** Predictive performance of SNAP‐25 for DNR.
**Table S3:** Bootstrap analysis for model performance.
**Table S4:** Incidence of DNR in high and low CSF SNAP‐25 groups.
**Table S5:** Incidence of DNR in high and low plasma SNAP‐25 groups.

## Data Availability

The data that support the findings of this study are available from the corresponding author upon reasonable request.
