# Supplementary material for: Preoperative Plasma and Cerebrospinal Fluid SNAP‐25 Levels Predict Delayed Neurocognitive Recovery in Elderly Patients: A Prospective Observational Study
Source: CNS Neurosci Ther. 2026 Jul 9;32(7):e71028. doi: 10.1002/cns.71028 (PMC13347317; doi:10.1002/cns.71028)
Supplement: Supplementary file 1 — Table S1: Neuropsychological test scores on postoperative day 7 in the DNR and non‐DNR groups. Table S2: Predictive performance of SNAP‐25 for DNR. Table S3: Bootstrap analysis for model performance. Table S4: Incidence of DNR in high and low CSF SNAP‐25 groups. Table S5: Incidence of DNR in high and low plasma SNAP‐25 groups. [file CNS-32-e71028-s001.docx]

**Supplementary Table 1.** Neuropsychological test scores on postoperative day 7 in the DNR and non-DNR groups

| Test | D group (n=21) | ND group (n=65) | *P* value |
| --- | --- | --- | --- |
| MMSE | 25.0 (24.0-26.0) | 27.0 (26.0-28.0) | ＜0.001^***^ |
| SCWT (s) | 47 (34-64) | 37 (31-47) | 0.015^*^ |
| CDT | 3 (3-4) | 4 (3-4) | 0.017^*^ |
| TMT (s) | 153.0 (89.5-201.0) | 112 (87-148) | 0.097 |
| DST | 6.0 (5.5-6.5) | 8.0 (7.0-9.0) | <0.001^***^ |

*All continuous variables are presented as median with interquartile range (IQR). P values were calculated using the Mann–Whitney U test; P^*^ means P value < 0.05; P^***^ means P value < 0.001. D group is defined as patients who developed DNR; ND group is defined as patients who did not develop DNR. DNR, delayed neurocognitive recovery; MMSE, Mini-Mental State Examination; SCWT, Stroop Color Word Test; CDT, Clock Drawing Test; TMT, Trail Making Test; DST, Digit Span Test.*

**Supplementary Table 2.** Predictive performance of SNAP-25 for DNR

| SNAP-25 concentration | AUC (95% CI) | Cut-off value | Sensitivity | Specificity |
| --- | --- | --- | --- | --- |
| CSF | 0.805 (0.706-0.905) | 737.5 | 0.905 | 0.569 |
| Plasma | 0.727 (0.618-0.837) | 543.5 | 0.714 | 0.708 |

*AUC, area under the ROC curve; CI, confidence interval; Cut-off value, optimal threshold determined by the maximum Youden index. DNR, delayed neurocognitive recovery; SNAP-25, synaptosomal-associated protein 25; CSF, cerebrospinal fluid.*

**Supplementary Table 3.** Bootstrap analysis for model performance

| Bootstrap | AUC | 95% CI | Standard error |
| --- | --- | --- | --- |
| CSF | 0.794 | 0.693-0.889 | 0.05 |
| Plasma | 0.719 | 0.607-0.826 | 0.06 |

*AUC means the mean value of AUCs obtained from 1,000 bootstrap resampling iterations. AUC, area under the ROC curve; CI, confidence interval; CSF, cerebrospinal fluid.*

**Supplementary Table 4.** Incidence of DNR in high and low CSF SNAP-25 groups

| DNR occurrence | High SNAP-25 groups | Low SNAP-25 groups | χ2 | *P* value |
| --- | --- | --- | --- | --- |
| No | 37 | 28 | 14.388 | <0.001^***^ |
| Yes | 2 | 19 |  |  |

*Categorical variables were compared using the chi-square test or continuity-corrected chi-square test. P^***^ means P value < 0.001. CSF, cerebrospinal fluid; SNAP-25, synaptosomal-associated protein 25; DNR, delayed neurocognitive recovery.*

**Supplementary Table 5.** Incidence of DNR in high and low plasma SNAP-25 groups

| DNR occurrence | High SNAP-25 groups | Low SNAP-25 groups | χ2 | *P* value |
| --- | --- | --- | --- | --- |
| No | 42 | 23 | 8.361 | 0.004^**^ |
| Yes | 6 | 15 |  |  |

*Categorical variables were compared using the chi-square test or continuity-corrected chi-square test. P^**^ means P value < 0.01. SNAP-25, synaptosomal-associated protein 25; DNR, delayed neurocognitive recovery.*
